# Targeted Drug Delivery for the Treatment of Abdominal Pain in Chronic Pancreatitis: A Retrospective Case Series

**DOI:** 10.7759/cureus.57285

**Published:** 2024-03-30

**Authors:** Guy P Cooper, Victor Progar, Kelly Grott, Feenalie Patel, Jackie Mon, Benjamin Bick, Timothy D Kelly, Raheleh Rahimi Darabad

**Affiliations:** 1 Anesthesia, Indiana University School of Medicine, Indianapolis, USA; 2 Gastroenterology and Hepatology, Indiana University School of Medicine, Indianapolis, USA; 3 Emergency Medicine, Indiana University School of Medicine, Indianapolis, USA

**Keywords:** targeted drug delivery, intrathecal pump therapy, severe pancreatitis, intrathecal opioid pump, abdominal pain, chronic pancreatitis

## Abstract

Summary: Abdominal pain secondary to chronic pancreatitis (CP) is difficult to manage and often requires chronic oral opioid therapy (OOT). Targeted drug delivery (TDD) allows for a diminished dose of opioid intake and improved pain levels. TDD has been used in different pain syndromes with only limited reports in CP.

Objective: The objective of this article is to perform a retrospective review of CP patients treated with TDD versus OOT to compare chronic pain control and consumed morphine-equivalent doses.

Methods: Patients receiving TDD between September 2011 and August 2018 were included. All patients were weaned off oral opioids one week before intrathecal trial and pump implantation. Patients with intrathecal trials providing at least 50% pain relief underwent pump implantation. Data were collected while on OOT and at two weeks, three months, and nine months post-implant. Data were analyzed with Microsoft Excel 365 MSO using means and standard deviations. P-values were calculated using a two-tailed student’s t-test with paired two-sample means.

Results: Twenty-three patients were analyzed. Pre-trial average pain score was 6.5/10 with a mean improvement with trials greater than 71%. The mean chronic baseline oral morphine milligram equivalents (MME) was 188. The mean MME on TDD at two weeks (0.36), three months (1.39), and nine months (2.47) were significantly lower than OOT. Mean pain scores were 6, 4.9, and 5.6 at two weeks, three months, and nine months, respectively, compared to 6.5 on OOT.

Discussion: The results of this study indicate that TDD provides improved pain control with significantly lower opioid doses.

## Introduction

Chronic pancreatitis (CP) is an inflammatory condition characterized by severe abdominal pain and both exocrine and endocrine pancreatic insufficiency. In the United States, pancreatitis is responsible for a significant cost burden estimated at 3.7 billion dollars per year [1], and the incidence of CP is increasing [2]. The cardinal symptom of CP is abdominal pain that is thought to be secondary to both neuropathic and inflammatory etiologies [3]. It is clinically challenging to manage due to its chronic nature and lack of response to non-opioid therapies. The frequency of attacks and the amount of pain have been associated with a decreased quality of life [4]. Multimodal treatment strategies include medications, endoscopic intervention, and, in intractable cases, surgical intervention. Total pancreatectomy with islet cell transplantation offers a definitive treatment of abdominal pain but carries with it an increased risk of mortality and increased risk of insulin dependence [5]. Targeted drug delivery (TDD) is a relatively new alternative therapy in CP patients.

The use of TDD allows for lower systemic opioid dosage and prevents patients from having to undergo surgery. While TDD has been used in cancer and chronic pain conditions for years, there are very few studies that evaluate its use in CP patients and none that look at long-term outcomes. A case series demonstrated in 2009 that TDD led to a significant decrease in pain with a morphine-equivalent dosage less than that required before pump placement. More recently, in 2016, Mokadem et al. published a case-control study that compared treatment with TDD to total pancreatectomy with islet cell transplant (TP + ICT), which demonstrated comparable pain control between the two groups with a significantly decreased risk of insulin dependence in the TDD group [5]. We aimed to perform an institutional retrospective review of CP patients treated with TDD versus oral analgesia to evaluate long-term pain control.

## Materials and methods

This retrospective case study was approved by the institutional review board committee of Indiana University School of Medicine. The study was performed at a single institution examining intrathecal opioid trials and permanent pump implants placed by three physicians between September 2011 and August 2018. The inclusion criteria were a diagnosis of CP and age greater than 18 years. The exclusion criteria were patients without baseline, two-week, three-month, or nine-month data.

Patients were weaned off oral opioids one week before the trial of the TDD. The trial was performed in an office with a single intrathecal opioid dose. Patients were allowed to restart oral opioids after the trial but were once again weaned one week before intrathecal pump implantation. Intrathecal catheter placement was performed at the T6 level. After implantation, patients were started on intrathecal opioid monotherapy without the use of any adjuvants. Morphine, hydromorphone, and fentanyl were utilized with conversion rates of 1, 4, and 15, respectively. Pumps were initiated on a standard minimum dosing, titrated to optimize pain reduction, and included continuous rate with or without automatic or patient-administered bolus-dosing regimens. Reported total daily morphine milligram equivalents (MME) reflect the 24-hour basal plus scheduled boluses or simple continuous rate. Pain scores and patient-reported pain relief were collected on oral opioid therapy (OOT) before weaning, immediately pre-trial, and two weeks, three months, and nine months post-implant.

The primary outcome was MME after intrathecal pump placement. Secondary outcomes included a numeric pain rating scale (1-10), adverse post-implant symptoms including constipation, pruritus, and nausea/vomiting, as well as post-implant pump complications including pump failure and/or need for pump removal. Demographics, symptomology, etiology, comorbidities, prior interventions, type, and intrathecal pump regimen data were collected and stored in a Redcap database and analyzed with Microsoft Excel 365 MSO. Continuous data were tabulated using means and standard deviations, and categorical data were listed according to frequency and percentile. P-values were calculated using a two-tailed student’s t-test with paired two-sample for means. P-values less than 0.05 were considered to be significant.

## Results

Twenty-three patients were analyzed. Patient demographics are presented in Table 1. All patients had data points on oral medications before weaning, pre-trial, two weeks, and three months. Nine patients (39%) did not have nine-month pain scores.

**Table 1:** Demographics Categorical variables are presented as proportions/percentages. Continuous variables are presented as mean ± SD.

Demographics	Overall (N = 23)
Age (years)	47 ± 13
Race	
African-American	2/23 (9%)
White	21/23 (91%)
Gender	
Female	16/23 (70%)
Male	7/23 (30%)
Other	1/23 (4%)
Weight (kg)	79 ± 24
Height (cm)	162 ± 21

Patients had an average age of 47 years. Two identified as African-American (2/23, 9%), and 21 (21/23, 91%) identified as white. Sixteen patients identified as female (16/23, 70%) and seven as male (7/23, 30%). The mean weight was 79 kg (SD = 24 kg), and the mean height was 162 cm (SD = 21 cm). A summary of the collected medical history is presented in Table 2.

**Table 2:** Medical history Categorical variables are presented as proportions/percentages. Continuous variables are presented as mean ± SD. ERCP: Endoscopic retrograde cholangiopancreatography.

Symptomology	
Exocrine insufficiency before procedures	14/23 (61%)
Endocrine insufficiency requiring medications	6/23 (26%)
Weight loss	12/23 (52%)
Etiology	
Ethanol use	4/23 (17%)
Tobacco	15/23 (65%)
Idiopathic	4/23 (17%)
Hereditary	1/23 (4%)
Ductal obstruction	1/23 (4%)
Comorbidities	
Age-adjusted Charlson comorbidity score	2 ± 2
Anxiety	16/23 (70%)
Depression	17/23 (74%)
Prior interventions	
Pancreas resection/decompression surgery	5/23 (22%)
ERCP	17/23 (74%)
Biliary sphincterotomy	13/23 (57%)
Pancreatic sphincterotomy	14/23 (61%)
Pancreatic duct stent	16/23 (70%)
Type	
Recurrent acute pancreatitis	4/23 (17%)
Large-duct pancreatitis	6/23 (26%)
Small-duct pancreatitis	17/23 (74%)

Before the intervention, symptoms included weight loss (12/23, 52%), exocrine insufficiency (14/23, 61%), and endocrine insufficiency requiring medications (6/23, 26%). The likely etiology for CP varied, with four patients having a history of ethanol use (4/23, 17%), 15 with tobacco use (15/23, 65%), four idiopathic (4/23, 17%), one hereditary (1/23, 4%), and one with ductal obstructions (1/23, 4%). No patients were diagnosed with autoimmune or hypertriglyceridemia etiology. The patients had comorbidities with an average age-adjusted Charlson comorbidity score of 2. Anxiety was present in 16 patients (16/23, 70%), and depression was present in 17 (17/23, 74%). There were multiple prior interventions performed including pancreas resection or decompression surgery (5/23, 22%), endoscopic retrograde cholangiopancreatography (17/23, 74%), biliary sphincterotomy (13/23, 57%), pancreatic sphincterotomy (14/23, 61%), and pancreatic duct stent (16/23, 70%). Four patients had recurrent acute pancreatitis (4/23, 17%), six had large-duct pancreatitis (6/23, 26%), and 17 had small-duct pancreatitis (17/23, 74%).

Table 3 summarizes opioid-related systemic effects throughout the study duration.

**Table 3:** Opioid-related systemic effects Results are presented as proportions/percentages.

Opioid-related systemic effects	Baseline	2 weeks	1 month	3 months
Constipation	10/23 (43%)	7/23 (30%)	7/23 (30%)	5/23 (22%)
Pruritus	1/23 (4%)	1/23 (4%)	0/23 (0%)	0/23 (0%)
Nausea/Vomiting	19/23 (83%)	17/23 (74%)	14/23 (61%)	12/23 (52%)

Ten patients entered the treatment with constipation, and no other patients reported constipation during the treatment. One patient entered the trial with pruritus, and one reported new-onset pruritus during the treatment. Nausea was present in 19 patients (19/23, 83%) intermittently at baseline, and only one person (1/23, 4%) reported new-onset nausea during the study. Complications were present in four patients (4/23, 17%), four of which had a post-dural puncture headache (4/23, 17%), and one required a blood patch to resolve the post-dural headache (1/23, 4%). There were two revisions (2/23, 9%); one was performed for pocket irritation, and one was performed for catheter migration. Pump medications included hydromorphone, morphine, and fentanyl. The starting medication was hydromorphone for 20 patients (20/23, 87%), morphine sulfate for two (2/23, 9%), and fentanyl for one (1/23, 4%). No patients were dropped early from the study.

**Figure 1:**
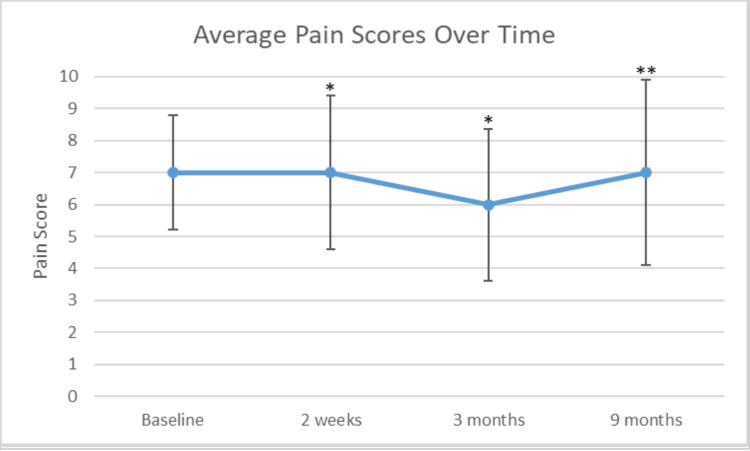
Average pain scores over time Results are presented as mean pain scores over time. Error bars represent a single standard deviation. * P-value < 0.05. ** P-value < 0.1.

Pre-trial average pain score was 6.5/10 with a mean improvement greater than 71%. The mean pain scores at two weeks, three months, and nine months post-TDD were 6, 4.9, and 5.6, respectively. A comparison was done of baseline chronic pain scores versus two-week, three-month, and nine-month intervals with p-values of 0.048, 0.003, and 0.096, respectively. These results are shown graphically in Figure 1. Four patients had complete resolution of their pain.

**Figure 2:**
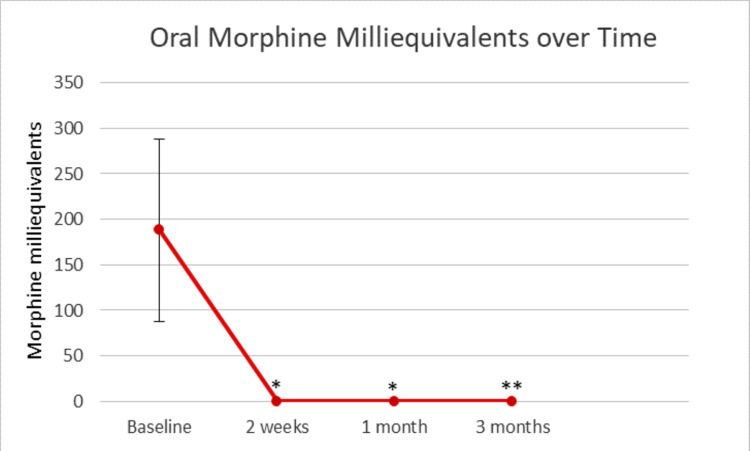
Oral morphine milliequivalents over time Error bars represent a single standard deviation. * P-value < 0.001. ** P-value < 0.005.

Before weaning, chronic baseline oral MME was 188. One patient had no recorded baseline MME. The mean intrathecal pump MME at two weeks, three months, and nine months post-TDD were 0.36, 1.39, and 2.47, respectively. Intrathecal MME was significantly lower at the two-week, three-month, and nine-month intervals compared to baseline pre-wean oral MME with respective p-values of 0.000495, 0.00052, and 0.0048. These results are shown graphically in Figure 2.

Four patients switched medications. One patient switched from morphine to hydromorphone, two switched from hydromorphone to morphine, and one switched from hydromorphone to fentanyl.

## Discussion

The objective of this study was to evaluate the potential benefit of intrathecal pain pumps for pain control compared to oral analgesics in patients with CP. The primary variables assessed were pain scores and oral MME requirement over time in relation to initiation of TDD. The principle finding of this study is that intrathecal pain pumps successfully decrease pain scores (on VAS 0-10) and decrease oral morphine equivalents required for pain control in patients with chronic abdominal pain secondary to CP (Figures 1, 2).

Specifically, this study demonstrated a significant reduction of pain (p-values < 0.05) at two weeks and three months relative to baseline. However, this reduction of pain was not seen at nine months. We suspect that medicine microdosing and inadequate optimization of the pain pump medication regimen likely contribute to this finding. Nonetheless, in two out of three timeframes, patients experienced less pain with the utilization of the intrathecal pain pump. It is possible that a more robust response would become apparent over a greater period and with a larger sample size.

While the reduction in pain scores pre- and post-TDD placement demonstrates a slight trend toward reduced pain, we discovered a more profound difference in the reduction of daily MME requirement for patients before and after starting intrathecal therapy. Before TDD placement, chronic pre-trial baseline oral MME was 188. The mean intrathecal pump MME at two weeks, three months, and nine months were 0.36, 1.39, and 2.47, respectively, reflecting a substantial reduction in daily MME requirement. This was not unexpected. TDD dosing delivers medication to the dorsal horn of the spinal cord, bypassing the blood-brain barrier and first-pass metabolism.

Delivery of TDD medication also impairs the transmission of noxious stimuli from propagating along the afferent pathway [6]. As a result, a lower effective dose can be used, and there are fewer opioid-related systemic effects, given less drug interaction with systemic receptors [7]. In our study, patients encountered few adverse effects traditionally associated with systemic opioid therapy [8-11]. In fact, over time, there were fewer reported incidents of constipation, pruritus, and nausea/vomiting than at baseline, possibly due to the directed administration of TDD medication, which avoids systemic receptors. Importantly, there was only a single episode of new-onset nausea.

The results of this study are encouraging, given the relative scarcity of studies on TDD in the management of CP. At present, the pathogenesis of abdominal pain in CP is still not completely understood. However, it is thought that both structural causes and altered central pain processing play a role. Historically, structural changes in the pancreas have been the focus of proposed pain mechanisms. These include strictures, fibrosis, and obstruction leading to increased intrapancreatic pressure, resultant ischemia, local pancreatic inflammation, and extrapancreatic manifestations [12-14]. However, studies have shown that morphological defects or metrics such as intrapancreatic pressures do not directly correlate with patient-reported symptoms [12,14]. Further, interventions aimed at relieving these purported structural sources of noxious stimuli such as stenting and surgery are not always effective in pain management [15]. It is now appreciated that structural causes of noxious stimuli are not sufficient to explain pain associated with CP. Hence, more attention has been directed to the role of altered central pain processing as well as peripheral and central sensitization [16-18]. Management of pain in patients with CP remains a challenge, and more scholarship is needed to elucidate the specific pain mechanisms and how to best utilize different treatment modalities.

There were significant limitations in this study. First, only 23 cases were included, which limits its generalizability to the general population. Second, the data was collected retrospectively and does not allow for prospective comparisons and/or analyses. Finally, a small minority of cases have incomplete data. Future investigations of TDD and CP would benefit from larger, randomized clinical trials.

## Conclusions

This study constitutes the largest study investigating the efficacy and safety of TDD in patients with CP, a debilitating disease with high-cost burden. Our data shows that TDD significantly improves pain levels in patients with chronic abdominal pain secondary to CP. It furthermore shows that TDD tremendously decreases the amount of opioid use in this patient population. TDD is an effective treatment in patients who have failed multiple other treatments including surgical interventions for the treatment of chronic abdominal pain. It poses a minimal risk of side effects and complications compared to OOT, with the most common complication reported as post-dural puncture headache. In summary, we suggest that TDD is an effective treatment for patients with chronic abdominal pain secondary to CP and should be considered to diminish opioid use and improve pain levels.
